# Aging exacerbates damage and delays repair of alveolar epithelia following influenza viral pneumonia

**DOI:** 10.1186/s12931-014-0116-z

**Published:** 2014-09-30

**Authors:** Lu Yin, Dahai Zheng, Gino V Limmon, Nicola HN Leung, Shuoyu Xu, Jagath C Rajapakse, Hanry Yu, Vincent TK Chow, Jianzhu Chen

**Affiliations:** Interdisciplinary Research Group in Infectious Diseases, Singapore-Massachusetts Institute of Technology Alliance for Research and Technology, Singapore, Singapore; Bioinformatics Research Center, School of Computer Engineering, Nanyang Technological University of Singapore, Singapore, Singapore; Department of Physiology, National University of Singapore, Singapore, Singapore; Institute of Bioengineering and Nanotechnology, Agency for Science, Technology and Research, Singapore, Singapore; Mechanobiology Institute, National University of Singapore, Singapore, Singapore; Interdisciplinary Research Group in BioSystems and Micromechanics, Singapore-Massachusetts Institute of Technology Alliance for Research and Technology, Singapore, Singapore; Department of Biological Engineering, Massachusetts Institute of Technology, Cambridge, MA USA; Host And Pathogen Interactivity Laboratory, Department of Microbiology, School of Medicine, National University Health System, National University of Singapore, Singapore, Singapore; The Koch Institute for Integrative Cancer Research and Department of Biology, Massachusetts Institute of Technology, 77 Massachusetts Avenue, 76-261, Cambridge, MA 02139 USA

**Keywords:** Influenza, Aged, Lung damage repair, Imaging analysis, Alveolar type I cell, Alveolar type II cell, Club cell, Pro-SPC-positive bronchiolar epithelial cell

## Abstract

**Background:**

Influenza virus infection causes significantly higher levels of morbidity and mortality in the elderly. Studies have shown that impaired immunity in the elderly contributes to the increased susceptibility to influenza virus infection, however, how aging affects the lung tissue damage and repair has not been completely elucidated.

**Methods:**

Aged (16–18 months old) and young (2–3 months old) mice were infected with influenza virus intratracheally. Body weight and mortality were monitored. Different days after infection, lung sections were stained to estimate the overall lung tissue damage and for club cells, pro-SPC^+^ bronchiolar epithelial cells, alveolar type I and II cells to quantify their frequencies using automated image analysis algorithms.

**Results:**

Following influenza infection, aged mice lose more weight and die from otherwise sub-lethal influenza infection in young mice. Although there is no difference in damage and regeneration of club cells between the young and the aged mice, damage to alveolar type I and II cells (AT1s and AT2s) is exacerbated, and regeneration of AT2s and their precursors (pro-SPC-positive bronchiolar epithelial cells) is significantly delayed in the aged mice. We further show that oseltamivir treatment reduces virus load and lung damage, and promotes pulmonary recovery from infection in the aged mice.

**Conclusions:**

These findings show that aging increases susceptibility of the distal lung epithelium to influenza infection and delays the emergence of pro-SPC positive progenitor cells during the repair process. Our findings also shed light on possible approaches to enhance the clinical management of severe influenza pneumonia in the elderly.

**Electronic supplementary material:**

The online version of this article (doi:10.1186/s12931-014-0116-z) contains supplementary material, which is available to authorized users.

## Background

Influenza A virus infection is a major global health challenge [1,2]. Although the virus infects people of all ages, elderly individuals (>75 years old) have more than 10-fold higher influenza-associated hospitalization and higher mortality compared to other age groups [3]. Studies have shown that a major contributing factor is the impaired immunity in the elderly including delayed viral clearance [4], delayed and diminished T-cell responses [5–8], poor immunological memory [5,6], and reduced cell-mediated immunity to vaccinations [9,10]. The delayed activation of adaptive immunity is associated with alterations in antigen-presenting cell activation in the elderly, leading to delayed production of cytokines and chemokines [4].

Impaired immunity is likely just one of the critical factors that render the elderly more vulnerable to influenza-associated morbidity and mortality. Influenza A virus infects many cell types in the lung, including club cells and alveolar type II cells (AT2s) [11,12]. Infected cells can be killed due to viral cytopathic effect. Immune responses to the virus-infected cells can also cause cell death and tissue damage. The loss of tissue integrity makes the lungs more susceptible to secondary bacterial infections. In many cases, by the time of hospitalization, the viral load is insignificant; morbidity and mortality of patients are likely caused by the severe lung tissue damage, pulmonary complications, and secondary bacterial pneumonia [13].

Given that the lungs are vital for survival, timely repair of lung damage following influenza virus infection is critical for recovery from the infection. Interestingly, evidence suggests that aging is associated with a functional decline of stem and progenitor cells [14–18], therefore compromising their roles in tissue maintenance and repair. For example, senescence-related changes in bone marrow-derived mesenchymal stem cells contribute to the higher susceptibility of lung fibrosis in the elderly [19]. BrdU-induced senescence of bronchiolar progenitor club cells results in impaired epithelial regeneration following naphthalene-induced bronchiolar damage [20]. However, the effect of aging on lung tissue damage and repair, i.e., the loss and regeneration of various lung epithelial cells, following influenza infection has not been fully characterized.

The lung comprises many types of epithelial cells that reside in anatomically distinct regions of the trachea, bronchioles and alveoli [21]. In the mouse, club (or Clara) cells, which express SCGB1A1, are the predominant cell type in the conducting airway, whereas alveolar epithelia are covered by AT1s and AT2s, expressing podoplanin (PDPN) and pro-surfactant protein C (pro-SPC), respectively. Club cells are known to exhibit progenitor cell properties to mediate the maintenance and repair of bronchiolar epithelia [22–24]. Recently, we and others show that club cells also give rise to AT1s and AT2s to repair alveolar epithelia following severe pulmonary damage [12,25,26]. We further show that club cell to AT2 differentiation goes through two successive intermediates. Bronchiolar epithelial cells that exhibit club cell morphology and express club cell marker, SCGB1A1, are first induced to express the AT2 cell marker, pro-SPC, following lung injury induced by influenza virus infection or bleomycin treatment. These cells, referred to as pro-SPC^+^ bronchiolar epithelial cells (or SBECs), then lose SCGB1A1 expression to give rise to pro-SPC^+^ cells in the damaged parenchyma [12,25].

In this study, we used automated image analysis [27] to quantify the damage and regeneration of club cells, AT1s, AT2s, and SBECs in young and aged mice following influenza infection. Our results show that although there is no difference in the extent of damage and kinetics of regeneration of club cells between young and aged mice, damage to AT1s and AT2s is more extensive, and regeneration of AT2s is significantly delayed in aged mice as compared to young mice. The delayed regeneration of AT2s is associated with a prolonged persistence of SBECs. We further show that oseltamivir treatment reduces viral load and lung damage, and promotes repair and recovery from infection in aged mice. These findings identify cellular defects during the repair of damaged lung tissue in aged mice, and shed light on possible approaches for better clinical management of severe influenza pneumonia in the elderly.

## Materials and methods

### Mice, influenza virus infection and sample preparation

Female C57BL/6 mice at 2–3 months and 8–9 months of age were purchased from Centre for Animal Resources, Singapore and housed in specific pathogen-free BSL2 facilities at National University of Singapore (NUS). The 2–3 month-old mice were allowed to acclimatize for about a week before they were used in experimentation and are referred to as “young mice”. The 8–9 month-old mice were allowed to age until they reached 16–18 months old, and are referred to as “aged mice”. Mice were anesthetized with ketamine (100 mg/kg body weight) and infected with influenza virus A/Puerto Rico/8/34 (H1N1) by intratracheal instillation (30 PFU/mouse). At the indicated days post-infection (dpi), mice were sacrificed and lung tissues were collected. The left lobes were fixed in 10% neutral buffered formalin solution (Sigma-Aldrich) overnight, processed with Tissue Processor (Leica, TP1020), and embedded in paraffin blocks. For each lobe, 20 transverse sections of 5 μm thicknesses were cut with microtome from the middle part of the lobe with 50 μm in between sections. Sections were mounted on polylysine-coated slides. Ten sections were used for hematoxilyn and eosin (H&E) staining, and 10 sections for immunofluorescence. The staining protocols are described in the online data supplement.

The right lobes were used for either lavage or virus plaque assay. The lavage was collected by flushing the right lobes with 1 ml of PBS twice. Cells were then centrifuged at 4000 rpm at 4°C (Thermal Fisher Scientific) and red blood cells were lysed with ACK lysing buffer (Life Technologies) for 90 sec. Cell numbers were counted under microscopy using a hemocytometer after staining with trypan blue (Sigma-Aldrich). To quantify virus titers in the lungs, the lobes were homogenized followed by centrifugation, and the supernatants were subjected to plaque assay. All animal experiments were approved by the Institutional Animal Care and Use Committee at NUS and Massachusetts Institute of Technology.

### Histological staining

Paraffin sections were de-waxed in Histo-Clear solutions twice (National Diagnostics) and rehydrated first in absolute ethanol three times, and then once each in 90% ethanol, 70% ethanol and 50% ethanol. H&E staining was processed according to standard protocol. For immunofluorescence staining, antigen retrieval was performed by incubating the lung sections with proteinase K solution (Sigma-Aldrich, 20 mg/ml proteinase K in 50 mM Tris-Cl, 1 mM EDTA, pH 8.0) at 37°C for 30 min. Sections were then blocked in blocking buffer (3% BSA, 0.2% Triton X-100 in PBS) for 1 hr. Polyclonal rabbit anti-CCSP (also known as SCGB1A1) antibody (US Biological, catalogue number C5828) was used at 1:100 dilution. Goat anti-pro-SPC antibody (Santa Cruz Biotechnology, catalogue number sc-7706) and goat anti-Podopanin antibody (R&D Systems, catalogue number AF3244) were used at 1:100 dilution. Incubation was performed at 4°C overnight in blocking buffer. Secondary Alexa Fluor 488-labeled donkey anti-rabbit antibody (Invitrogen, catalogue number A21206) and Alexa Fluor 546-labeled donkey anti-goat antibody (Invitrogen, catalogue number A11056) were used at 1:100 dilution. Incubation was performed at room temperature for 1 hr. Cover slips were mounted on stained sections with antifade reagent containing DAPI (Invitrogen).

### Image acquisition

MIRAX MIDI system (Carl Zeiss) equipped with bright field and fluorescence illumination was used to scan the stained lung sections. Whole-slide scanned images were captured with Axiocam MR(m) (Carl Zeiss), and converted to TIFF format with Miraxviewer software (3DHISTECH). The images used for quantification were down-sampled eight times from the original images to reduce the computation time and the use of memory space.

### Image analysis

The algorithms for computing infiltration index, club cell coverage index, AT2 relative density and percentage of SBEC-containing bronchioles are described previously [27]. Briefly, infiltration index is defined as the ratio of infiltrated areas to total alveolar area in H&E stained lung section images. To identify infiltrated areas with high nuclei density, an H&E image was converted to greyscale, and then subsampled by factor of two, four and eight respectively to form the image pyramid of subsampled images [28]. An intensity threshold was then applied to identify the dark-colour pixels (representing nuclei) in each level of the image pyramid. A pixel was eventually selected only if it was identified as dark-colour pixel in all levels of the image pyramids. These pixels were then dilated to form connected infiltrated areas. Total alveolar area was identified from the original image by using a size threshold to exclude large empty blood vessels and bronchioles in the lung sections. The infiltration index was then computed as the ratio of infiltrated areas to total alveolar area.

Club cell coverage index is the coverage density of SCGB1A1^+^ club cells on the interior wall of bronchioles in immunofluorescent images of lung sections. The boundaries of bronchiolar epithelia were identified by first using Delaunay triangulation [29] to determine clusters of boundary nuclei with long distance from neighbouring nuclei, then performing region growing from the centres of each cluster [30]. Based on SCGB1A1 signal at the identified boundaries of bronchiolar epithelia, all the bronchioles were clustered into high-club-cell-coverage ones (HC) and low-club-cell-coverage-ones (LC) using Ostu method [31]. The club cell coverage index was computed as the ratio of HC-to-LC frequency.

AT2 relative density measures the two dimensional density of AT2s in immunofluorescent images of lung sections. To compute it, K-means clustering [32] and watershed method [33] were used to separate healthy and damaged areas in the lung section and identify AT2s in the healthy areas based on SPC signal. The total number of AT2s was counted. The AT2 relative density was then computed as the number of AT2s in unit tissue area.

Percentage of SBEC-containing bronchioles among the total bronchioles is used to quantify the induction of SBECs. Bronchioles were identified using the same feature extraction method described in computing club cell coverage index. Damaged areas were identified using the same feature extraction method described in computing AT2 relative density. The SCGB1A1^+^ and SCGB1A1^−^ SBECs were then determined by checking the co-localization of SCGB1A1 and pro-SPC signals at bronchioles in the damaged areas. The number of total bronchioles and SBEC-containing bronchioles were then counted respectively. The percentage of SCGB1A1^+^ and SCGB1A1^−^ SBEC-containing bronchioles were computed by calculating the percentage of SCGB1A1^+^ and SCGB1A1^−^ SBEC-containing bronchioles to total bronchioles, respectively.

An additional automated computer algorithm was developed in this study to compute AT1 coverage index as the ratio of alveolar area covered with PDPN^+^ AT1s to total alveolar area. Total alveolar area was identified by manual thresholding DAPI fluorescence intensities in immunofluoresecnt images, and dilating using “disk” operator with 5 pixels in size to create the mask of the tissue, followed by filtering of small areas less than 10000 pixels, filling holes, and eroding back with same operator and dimension (Additional file 1: Figure S1). The alveolar area covered with PDPN^+^ AT1s was segmented in a similar way by manual thresholding PDPN fluorescence intensity, and then performing the same dilation, filtering, hole-filling and erosion process. All image processing and computation algorithms were implemented using Matlab with image processing toolbox (Math Works, Inc.). The Matlab codes are available on request.

### Statistical analysis

Student t-test was used to determine the statistical significance of body weight, infiltration index, AT1 coverage index, AT2 relative density, club cell coverage index, percentage of total SBEC-containing bronchioles, percentage of SCGB1A1^+^ and SCGB1A1^−^ subsets between aged and young mice, and aged mice with and without oseltamivir treatment. Student t-test was also used to determine the statistical significance of viral load in the lung between oseltamivir-treated and untreated aged mice. One-way ANOVA was used to compare the kinetic profiles of club cell coverage index between aged and young mice. All statistics were computed with Matlab (Math Works, Inc.).

## Results

### Influenza virus infection causes more severe lung disease in aged mice

**Figure 1:**
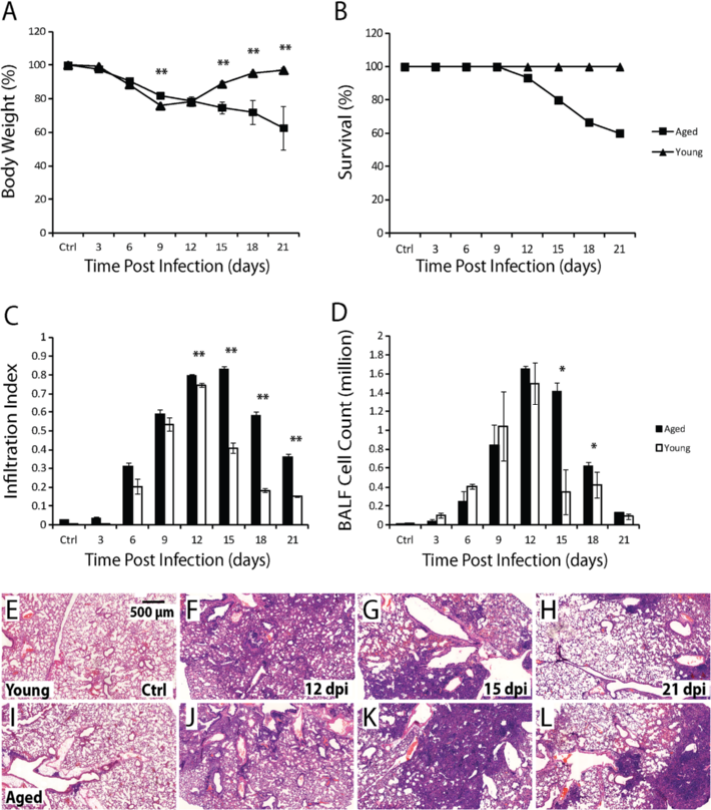
**Influenza virus infection causes more severe pulmonary disease in aged mice. A**. Body weight change in aged (square) and young (triangular) mice following influenza infection. **B**. Survival in aged (square) and young (triangular) mice following influenza infection. N=15 for both aged and young mice. **C**. Infiltration index in aged (solid column) and young (open column) mice following influenza infection. Ten aged and 10 young mice were used for each time-point. **D**. Cell number in BALF (after lysis of RBC) in aged (solid column) and young (open column) mice following influenza infection. Five aged and 5 young mice were used for each time-point. All error bars represent standard errors. *p<0.05, **p<0.01 by Student t-test. **E-H**. Representative H&E images of lung sections of young mice before infection **(E)**, and at 12 **(F)**, 15 **(G)**, and 21 **(H)** dpi. **I-L**. Representative H&E images of lung sections of aged mice before infection **(I)**, and at 12 **(J)**, 15 **(K)**, and 21 **(L)** dpi. Scale bar: 500 μm.

Female C57BL/6 mice aged 2–3 months (young mice) and 16–18 months (aged mice) were infected with influenza virus A/Puerto Rico/8/34 (H1N1, PR8) by intratracheal instillation (30 PFU/mouse). Body weight and survival of the mice were monitored. Although the young mice lost up to ~24% of the body weight by 9 days post infection (dpi), they regained their body weight completely around 18 dpi (Figure 1A). In contrast, the aged mice continuously lost weight and by 21 dpi the body weight was reduced by ~37%. Furthermore, the sublethal challenge dose of influenza virus in the young mice caused significant mortality in the aged mice, which started to die at 12 dpi, and ~40% were dead by 21 dpi (Figure 1B). Thus, influenza virus infection causes more severe disease in the aged mice.

To quantify lung damage and repair, lungs were harvested before infection (control) and at 3, 6, 9, 12, 15, 18, 21 dpi, stained with H&E, and analysed for inflammatory cell infiltration. The infiltration index is defined as the ratio of infiltrated areas to total alveolar areas in H&E images of lung sections (see Materials and methods for more detail). In young mice, infiltration in the alveolar region was detected 6 dpi and reached the peak level of ~75% of alveolar region 12 dpi. Thereafter, infiltrating cells disappeared rapidly and only ~15% of the alveolar region was still infiltrated by 21 dpi (Figure 1C, E-H). In the aged mice, the kinetics of initial infiltration was similar to that in young mice. However, the peak level of infiltration in the aged mice was significantly higher (~80%) at 12 dpi and persisted until 15 dpi. In addition, a much slower reduction was observed thereafter, i.e. ~36% of the alveolar region was still infiltrated by 21 dpi (Figure 1C, I-L). Inflammatory cell infiltration was also assayed by counting the number of nucleated cells in the bronchioalveolar lavage fluid (BALF). Although cell numbers in both aged and young mice reached the peak level (1.5 × 10^6^) at 12 dpi, approximately 5-fold more cells were recovered in the BALF from the aged mice than the young mice at 15 dpi (Figure 1D). Thus, compared to the young mice, influenza virus infection also causes prolonged inflammatory cell infiltration in the lungs of the aged mice, consistent with the more severe disease.

### Aged mice sustain more extensive loss of AT1 and AT2 cells, and delayed recovery of AT2s

To compare damage and repair of bronchiolar and alveolar epithelia between young and aged mice, we quantified the changes of club cells, AT1s and AT2s over time by calculating the club cell coverage index, AT1 coverage index and AT2 relative density [27]. All three indices were based on analysis of immunofluorescent images of lung sections (see Materials and methods for details) in order to preserve spatial relationships among different cell types in the tissue.

**Figure 2:**
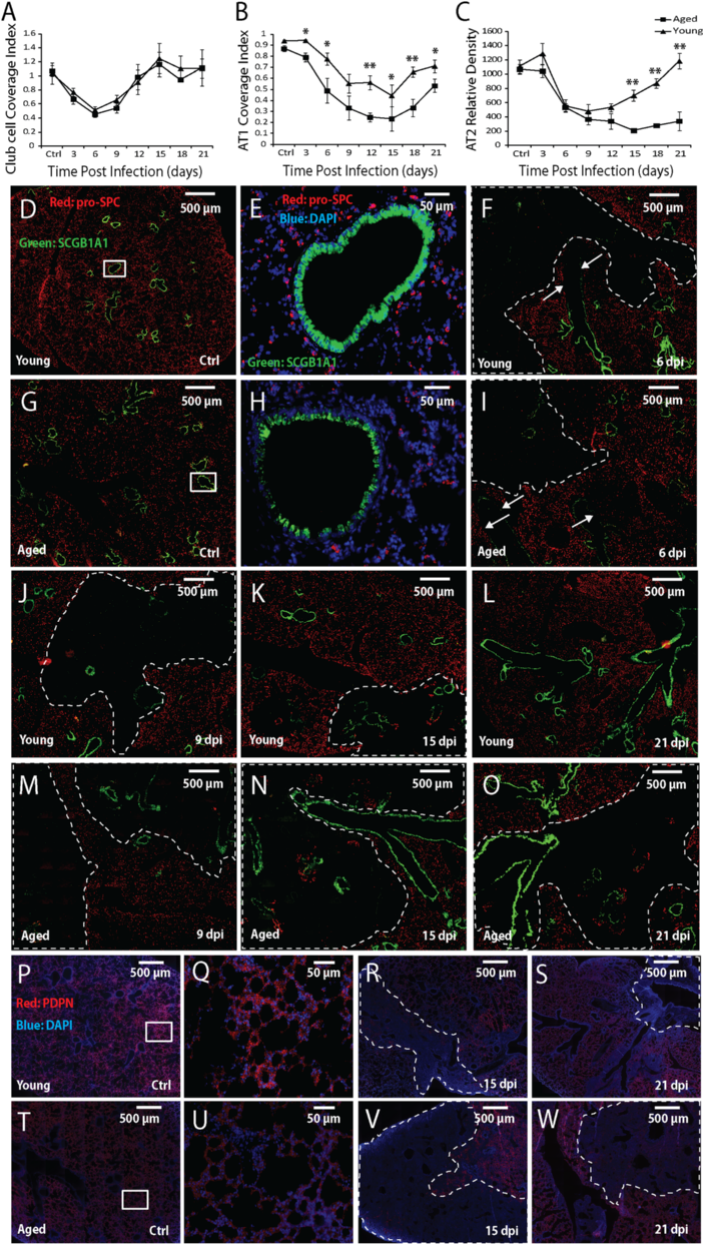
**Comparison of loss and recovery of club cells, AT1s and AT2s between aged and young mice following influenza pneumonia. A-C**. Club cell coverage index **(A)**, AT1 coverage index **(B)**, and AT2 density (number per mm^2^ tissue area) **(C)** in aged (square) and young (triangular) mice following influenza infection. All error bars represent standard errors. *p < 0.05, **p < 0.01 by Student t-test. **D-W**. Representative immunofluorescence staining of lung sections for SCGB1A1 (green) and pro-SPC (red) in D-O (DAPI stain is shown in blue in E and H), and PDPN (red) and DAPI (blue) in P-W at the indicated time-points following influenza infection. The boxed areas in D, G, P and T are shown in higher magnification in E, H, Q and U, respectively. The arrows point to loss of SCGB1A1-expressing club cells; in D-O the dotted lines circle the regions with loss of pro-SPC-expressing AT2s; in P-W the dotted lines circle the regions with loss of PDPN-expressing AT1s . Scale bars are 500 μm for the low magnification images, and 50 μm for the high magnification images. Ten aged and 10 young mice were used for each time-point.

Club cell coverage index is the density of club cells on the interior wall of bronchioles. In healthy lungs, the majority of bronchiolar epithelium was covered by SCGB1A1^+^ club cells (Figure 2D, E, G, H). The biggest loss of club cells was observed at 6 dpi, and the full recovery was completed by 15 dpi in both aged and young mice (Figure 2A, F, I, K, N). No significant difference in club cell coverage index was observed at any time-point following infection between aged and young mice (*p* > 0.05 by one-way ANOVA), indicating that loss of club cells and regeneration of club cells following infection were similar between aged and young mice.

AT1 coverage index is the ratio of AT1-covered alveolar areas to total alveolar areas. In healthy lungs, over 95% of alveolar epithelial surface was covered by PDPN^+^ AT1s (Figure 2P, Q, T, U) [34], with pro-SPC^+^ AT2s scattered uniformly (Figure 2D, E, G, H). Following influenza infection, AT1 coverage decreased in both aged and young mice (Figure 2B, R, V). However, the magnitude of AT1 cell loss was significantly larger in the aged mice than the young mice at all time-points except 9 dpi. AT1 coverage increased from 15 to 18 and 21 dpi (Figure 2B, S, W), but the similar difference was maintained between the aged and young mice, suggesting similar kinetics of AT1 regeneration in both age groups.

AT2 relative density is the number of pro-SPC^+^ AT2s per unit tissue area. Before infection, AT2 relative density was ~1100 cells per mm^2^ tissue area in both aged and young mice. The density decreased following infection, and reached ~480 cells/mm^2^ at 9 dpi in young mice (Figure 2C, J). Subsequently, AT2 density recovered gradually and reached the pre-infection level by 21 dpi in the young mice (Figure 2C, L). In contrast, AT2 relative density in the aged mice decreased continuously to ~210 cells/mm^2^ by 15 dpi (Figure 2C, N). Even at 21 dpi, the AT2 density was only ~340 cells/mm^2^ (Figure 2C, O). These results clearly show that compared to young mice, aged mice lose more AT2s and exhibit delayed AT2 regeneration following influenza virus pneumonia.

### The induction of SBECs is reduced but the duration is prolonged in aged mice

**Figure 3:**
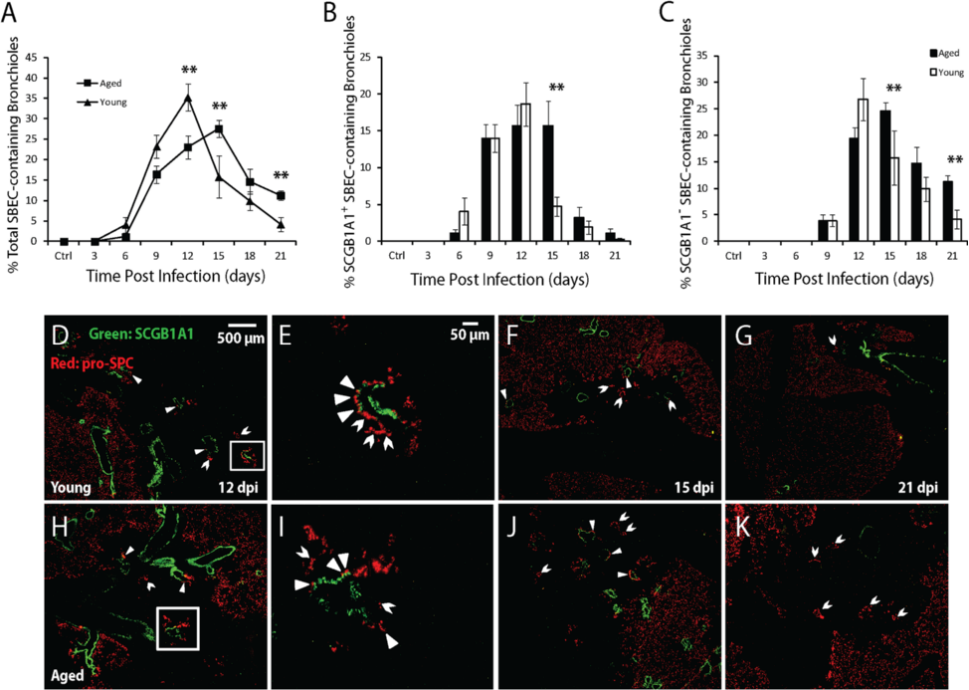
**Induction of SBECs in aged and young mice following influenza pneumonia. A**. The percentage of total SBEC-containing bronchioles (both SCGB1A1^+^ and SCGB1A1^−^ subset) in aged (square) and young (triangular) mice following influenza infection. **B**. The percentage of SCGB1A1^+^ SBEC-containing bronchioles in the aged (solid column) and the young (open column) mice. **C**. The percentage of SCGB1A1^−^ SBEC-containing bronchioles in the aged (solid column) and the young (open column) mice. Ten aged and 10 young mice were used for each time-point. All error bars represent standard errors. *p < 0.05, **p < 0.01 by Student t-test. **D-K**. Representative immunofluorescent staining of lung sections for SCGB1A1 (green) and pro-SPC (red) at the indicated time-points following influenza infection (DAPI staining not shown). The boxed areas in D and H are shown at higher magnification in E and I, respectively. Solid and open arrowheads point to SCGB1A1^+^ and SCGB1A1^−^ SBECs, respectively. Scale bars are 500 μm for the low magnification images, and 50 μm for the high magnification images.

The similar loss and regeneration of club cells between young and aged mice, but delayed regeneration of AT2s in aged mice suggests delayed club cell to AT2 differentiation in aged mice following influenza viral pneumonia. Following infection, club cells are induced to differentiate into AT2s via SBECs [12,25], and we therefore compared the induction of SBECs [27] between aged and young mice. The induction of SBECs is quantified as the percentage of SBEC-containing bronchioles among the total bronchioles, measured in immunofluorescent images of lung sections (see Materials and methods for details). In both groups of animals, SBECs (pro-SPC^+^ cells in the bronchiolar epithelia) started to appear at 6 dpi (Figure 3A). In the young mice, the percentage of total SBEC-containing bronchioles (both SCGB1A1^+^ and SCGB1A1^−^ subsets) peaked at 12 dpi with ~35% of total bronchioles containing SBECs (Figure 3A, D). However, in the aged mice, the percentage of total SBEC-containing bronchioles peaked later at 15 dpi and with a significantly lower percentage (~28%) (Figure 3A, J). After reaching the peak level at 12 dpi, the percentage of total SBEC-containing bronchioles decreased rapidly in young mice and was only ~4% by 21 dpi (Figure 3A, G). In contrast, the decrease was slower in the aged mice, and ~12% of bronchioles still contained SBECs by 21 dpi (Figure 3A, K).

We further quantified SCGB1A1^+^ and SCGB1A1^−^ subsets of SBECs separately. In both the aged and young mice, SCGB1A1^+^ SBECs started to appear at 6 dpi followed by SCGB1A1^−^ SBECs at 9 dpi (Figure 3B). In young mice, the percentage of SCGB1A1^+^ SBEC-containing bronchioles peaked at ~19% by 12 dpi, followed by a rapid decrease, and was almost non-detectable by 21 dpi (Figure 3B, D-G). In aged mice, the peak percentage of SCGB1A1^+^ SBEC-containing bronchioles was slightly lower at ~16%, but lasted longer from 12 to 15 dpi before a rapid decrease to ~4% by 21 dpi (Figure 3B, H-K). In young mice, the percentage of SCGB1A1^−^ SBEC-containing bronchioles peaked at ~27% at 12 dpi, and gradually decreased to ~4% by 21 dpi (Figure 3C, D-G). In aged mice, the percentage of SCGB1A1^−^ SBEC-containing bronchioles peaked at a similar level (~24%) but later at 15 dpi, and remained higher (~11%) by 21 dpi (Figure 3C, H-K), consistent with the sustained levels of SCGB1A1^+^ SBECs from 12 to 15 dpi. These results indicate that differentiation from club cells to AT2s is delayed at the intermediate stage of SBECs in the aged mice.

### Oseltamivir treatment of the aged mice reduces damage to club cells, AT1s, AT2s and SBECs

**Figure 4:**
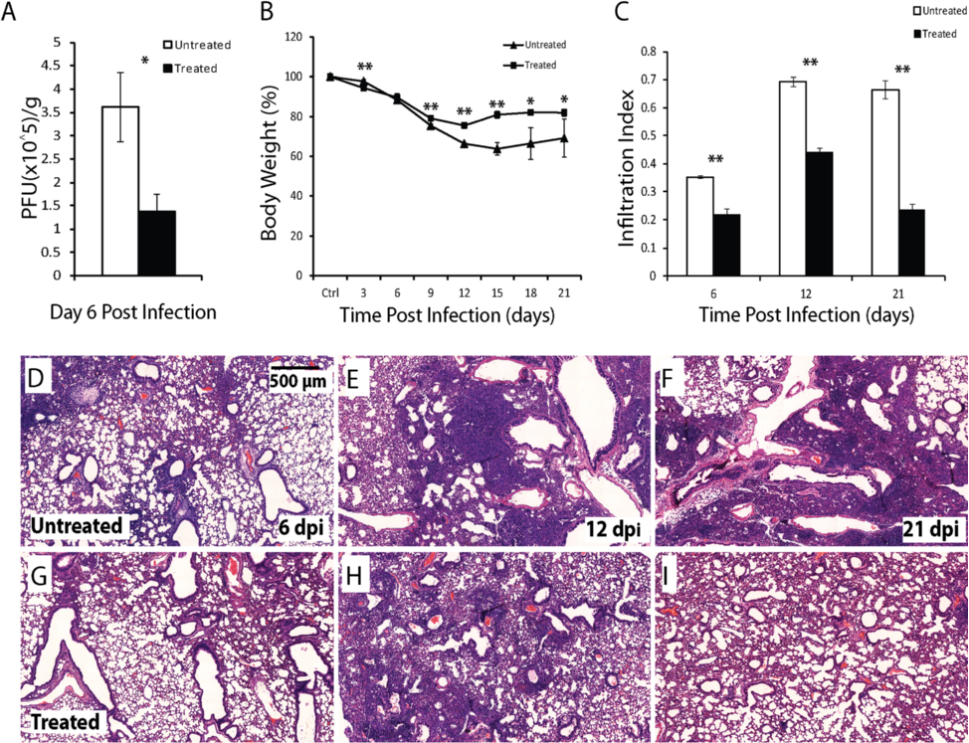
**Oseltamivir treatment reduces disease severity in the aged mice following influenza infection. A**. Body weight change in the aged mice with (square) and without (triangular) oseltamivir treatment following influenza infection. **B**. Viral load (PFU/g of lung tissue) in the aged mice with (solid column) and without (open column) oseltamivir treatment following influenza infection. **C**. Infiltration index in the aged mice with (solid column) and without (open column) oseltamivir treatment following influenza infection. Five oseltamivir-treated and 5 untreated aged mice were used for each time-point. All error bars represent standard errors. *p < 0.05, **p < 0.01 by Student t-test. **D-F**. Representative H&E images of lung sections of the aged mice without oseltamivir treatment at 6 **(D)**, 12 **(E)**, and 21 **(F)** dpi. **G-I**. Representative H&E images of lung sections of the aged mice with oseltamivir treatment at 6 **(G)**, 12 **(H)**, and 21 **(I)** dpi. Scale bar: 500 μm.

We investigated the effect of the anti-influenza agent oseltamivir on lung damage and regeneration. Aged mice were infected intratracheally, and given PBS (untreated control) or oseltamivir (150 mg/kg body weight) intraperitoneally every 12 hrs for 5 days starting at 2 dpi. Mice were monitored for body weight every 3 days, and euthanized for measuring viral load at 6 dpi and for lung histology at 6, 12 and 21 dpi. As expected, oseltamivir treatment reduced the virus titer in the lung by ~2 fold at 6 dpi (Figure 4A). Oseltamivir-treated mice also lost less weight and started to recover earlier (Figure 4B). Inflammatory cell infiltration into the lungs (Figure 4C) at 6, 12, and 21 dpi was significantly diminished in the oseltamivir-treated mice (Figure 4G-I) than in untreated aged mice (Figure 4D-F).

**Figure 5:**
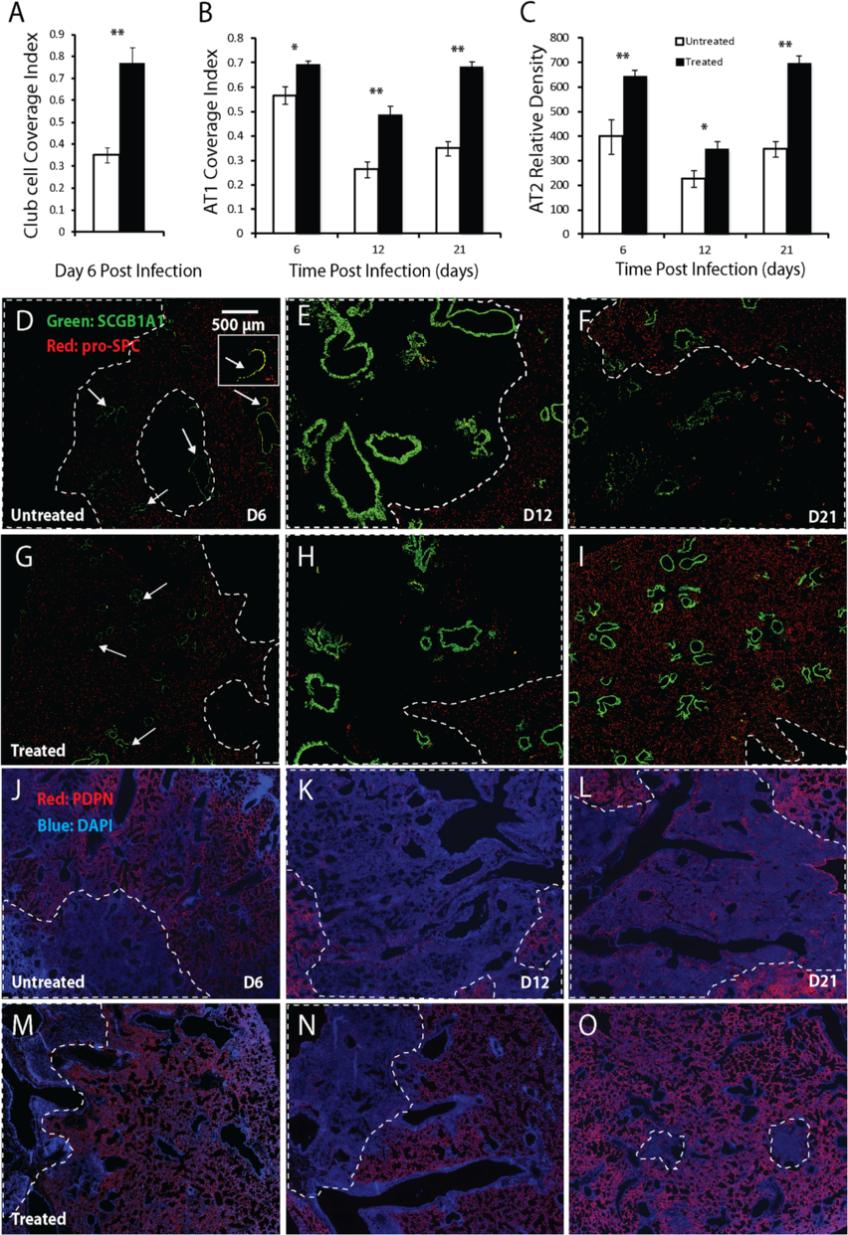
**Comparison of club cell, AT1, and AT2 coverage in oseltamivir-treated and untreated aged mice following influenza infection. A-C**. Club cell coverage index **(A)**, AT1 coverage index **(B)**, and AT2 density (number per mm^2^ tissue area) **(C)** in the aged mice with (solid column) and without (open column) oseltamivir treatment following influenza infection. Five oseltamivir-treated and 5 untreated aged mice were used for each time-point. All error bars represent standard errors. *p < 0.05, **p < 0.01 by Student t-test. **D-O**. Representative immunofluorescence staining of lung sections for SCGB1A1 (green) and pro-SPC (red) in D-I (DAPI stain is not shown), and PDPN (red) and DAPI (blue) in J-O at 6, 12 and 21 dpi. The arrows point to loss of SCGB1A1-expressing club cells; the insert in D shows a magnified view of a bronchiole with club cell loss; in D-I the dotted lines circle the regions with loss of pro-SPC-expressing AT2s; in J-O the dotted lines circle the regions with loss of PDPN-expressing AT1s. Scale bar: 500 μm.

**Figure 6:**
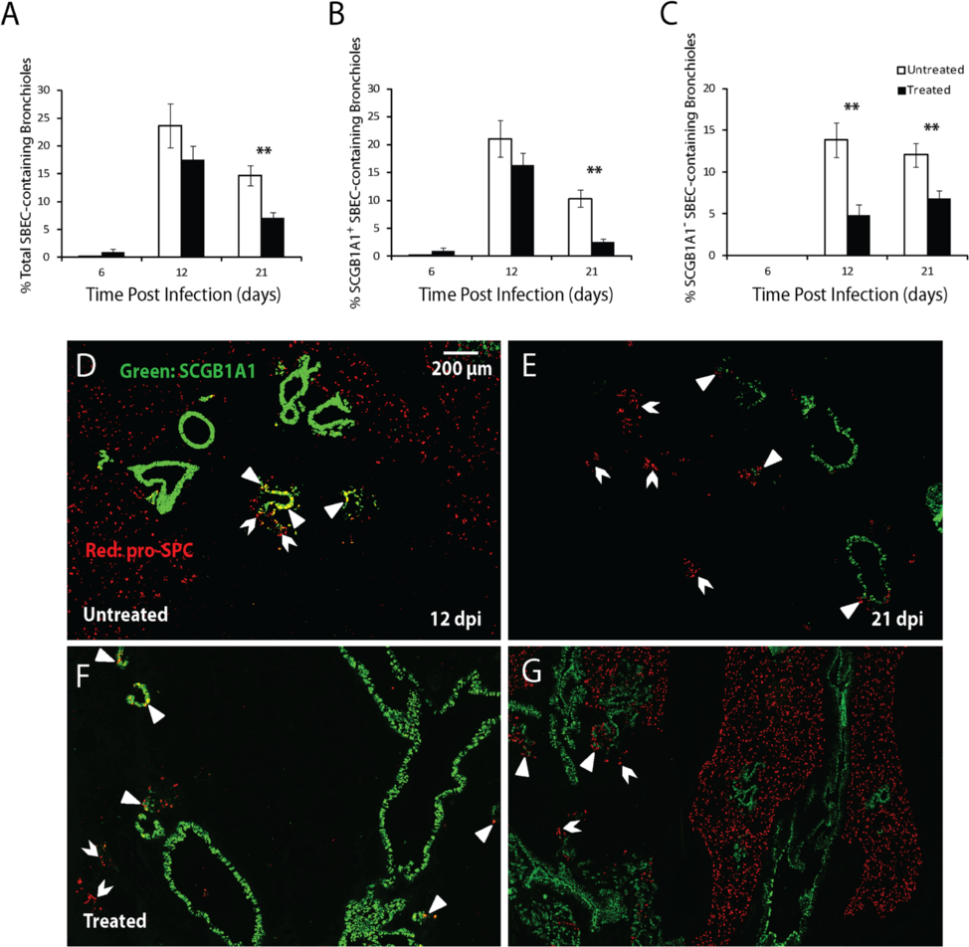
**Induction of SBECs in aged mice with and without oseltamivir treatment following influenza infection. A-C**. The percentages of total SBEC- **(A)**, SCGB1A1^+^ SBEC- **(B)**, and SCGB1A1^−^ SBEC-containing bronchioles **(C)** in the aged mice with (solid column) and without (open column) oseltamivir treatment following influenza infection. Five oseltamivir-treated and 5 untreated aged mice were used for each time-point. All error bars represent standard errors. *p < 0.05, **p < 0.01 by Student t-test. **D-G**. Representative immunofluorescence staining of lung sections for SCGB1A1 (green) and pro-SPC (red) at the indicated time-points following influenza infection (DAPI stain was not shown). Scale bars: 500 μm.

Corresponding to the reduced disease severity following oseltamivir treatment, club cell coverage index (Figure 5A), AT1 coverage index (Figure 5B) and AT2 relative density (Figure 5C) at 6 dpi were all significantly higher in the treated mice (Figure 5G-I, 5 M-O) than in untreated mice (Figure 5D-F, 5 J-L). Similarly, significantly higher levels of AT1 coverage index and AT2 relative density were observed in the treated mice at 12 and 21 dpi. The induction of SBECs, SCGB1A1^+^ and SCGB1A1^−^ subsets was significantly lower in the treated mice than in untreated mice at 21 dpi (Figure 6). At 12 dpi, although the percentage of total SBEC-containing bronchioles, SCGB1A1^+^ and SCGB1A1^−^ subsets were lower in the treated mice than in untreated mice, the difference was only significant for SCGB1A1^−^ subset. Thus, the reduced disease severity following oseltamivir treatment of the aged mice is correlated with decreased damage of club cells, AT1s, AT2s and SBECs.

## Discussion

Following influenza virus infection, elderly patients suffer from a much higher rate of hospitalization and mortality than individuals from other age groups [3,35–38]. Similar to humans, influenza virus infection also induces much more severe disease in aged mice than young mice. Compared to young mice which exhibited only transient weight loss following influenza pneumonia, the aged mice continued to lose weight even at 21 dpi. The sublethal dose of influenza H1N1 virus in the young mice led to 40% mortality of the aged mice by 21 dpi. The more severe disease in the aged mice correlated with higher and prolonged inflammatory cell infiltration in the lung and in the BALF, consistent with previous reports [4,7,8].

Reproducing the increased morbidity and mortality in the aged mice provides a model system to investigate the effect of aging in lung damage and repair following influenza infection at the cellular level. We compared the damage of major lung cell types, including club cells, AT1s, AT2s and SBECs, and monitored their recovery over time between young and aged mice. We used histological analysis because it preserves spatial relationships among different cell types in the tissue [27]. In addition, loss of histological staining has been associated with loss of specific cell types. For example, dead or dying club cells slough off into the airway lumen, resulting in the loss of SCGB1A1 expression along the bronchiolar epithelia [12,27,39]. Loss of PDPN expression is associated with loss of AT1s in many lung injury models [40,41]. Similarly, loss of pro-SPC expression following influenza infection is due to depletion of AT2s as indicated by genetic tracing in rCCSP-rtTA:tetO-Cre:ACTB-mT-EGFP transgenic mice [12]. Thus, the damage and recovery of club cells, AT1s and AT2s can be quantified based on the expression of their respective protein markers in lung sections [27].

We show that loss and regeneration of club cells in bronchiolar epithelia were similar in aged and young mice following influenza pneumonia. Club cells are known to possess self-renewal capacity in response to bronchiolar damage [23,42], and a sub-population of club cells, referred to as variant club cells, are able to proliferate to give rise to more club cells in response to naphthalene-induced injury [42–44]. Our data suggest that aging does not affect club cell regeneration during the repair of influenza-induced damage of bronchiolar epithelia.

Our results show that the loss of AT1s was more severe in the aged than the young mice (Figure 2B). However, the kinetics of AT1 regeneration appeared to be similar between the two groups because the same difference in the level of AT1s was maintained during their recovery. The loss of AT2s was also significantly more severe in the aged than the young mice (Figure 2C). Furthermore, the regeneration of AT2s was much slower in the aged mice as there was very little recovery even at 21 dpi. The delayed regeneration of AT2s compared to AT1s does not support the long-standing notion that AT2s give rise to AT1s during the repair of alveolar damage under many pathological conditions [45,46], but is consistent with recent findings that SCGB1A1^+^ bronchiolar cells could give rise to AT2s and AT1s during the repair of alveolar epithelia [12,25,26]. These results show that the aged mice suffer from much more severe damage of alveolar epithelia as well as delayed repair following influenza pneumonia.

We have previously shown that club cells differentiate into AT2s through SCGB1A1^+^ and then SCGB1A1^−^ SBECs [12,25]. Consistently, SBECs were induced in both aged and young mice between 9 to 15 dpi, after club cell regeneration (6–15 dpi), and before regeneration of AT2s and AT1s (15 dpi and beyond). Peak percentage of total SBEC-containing bronchioles was significantly lower in aged mice (15 dpi) than young mice (12 dpi) (p < 0.01), indicating impaired differentiation from club cells to SBECs (Figure 3A). In the aged mice, high levels of SBECs persisted much longer (Figure 3A), especially the SCGB1A1^−^ subset (Figure 3C), suggesting an impaired differentiation from SBECs to AT2s. These results reveal that aging affects multiple steps of alveolar epithelial repair, from club cells to SBECs to AT2s.

We investigated the effect of the anti-influenza agent oseltamivir on lung damage and repair in the aged mice. When administered after infection, oseltamivir reduced the viral load, inflammatory cell infiltration into the lung, and loss of club cells, AT1s and AT2s in the aged mice. Associated with the reduced lung damage, induction of SBECs was also more transient in the treated mice, indicating more efficient differentiation from club cells to AT2s. Consistently, oseltamivir-treated mice lost less weight and experienced lower mortality compared to untreated aged mice (2 out of 5 untreated aged mice died between 12 to 21 dpi, but none of the 5 treated aged mice died). These data support the strategy of mitigating influenza-associated morbidity and mortality by ameliorating lung damage via viral load reduction. Antiviral agents may achieve their therapeutic effect partly by mitigating lung injury and promoting timely repair.

The increased susceptibility of the elderly to influenza-induced morbidity and mortality as well as decreased responsiveness to influenza vaccination has traditionally been associated with impaired immunity [47,48]. Our study in the aged mice clearly shows that exacerbated lung damage and delayed repair also constitute a significant factor contributing to the greater disease severity. Our study also suggests that antiviral therapy may also confer beneficial effects by reducing tissue damage [49,50].

## Additional file

Additional file 1: Figure S1. Illustration of algorithm for computing AT1 coverage index A. A section of lung lobe stained with PDPN antibody (red) and DAPI (blue) B. Segmented nucleus channel from original image C. Segmented PDPN channel from original image D. Mask of total alveolar area E. Mask of PDPN + alveolar area.
