# Supplementary figures and images for: Aging exacerbates damage and delays repair of alveolar epithelia following influenza viral pneumonia
Source: Respir Res. 2014 Sep 30;15(1):116. doi: 10.1186/s12931-014-0116-z (PMC4189598; doi:10.1186/s12931-014-0116-z)

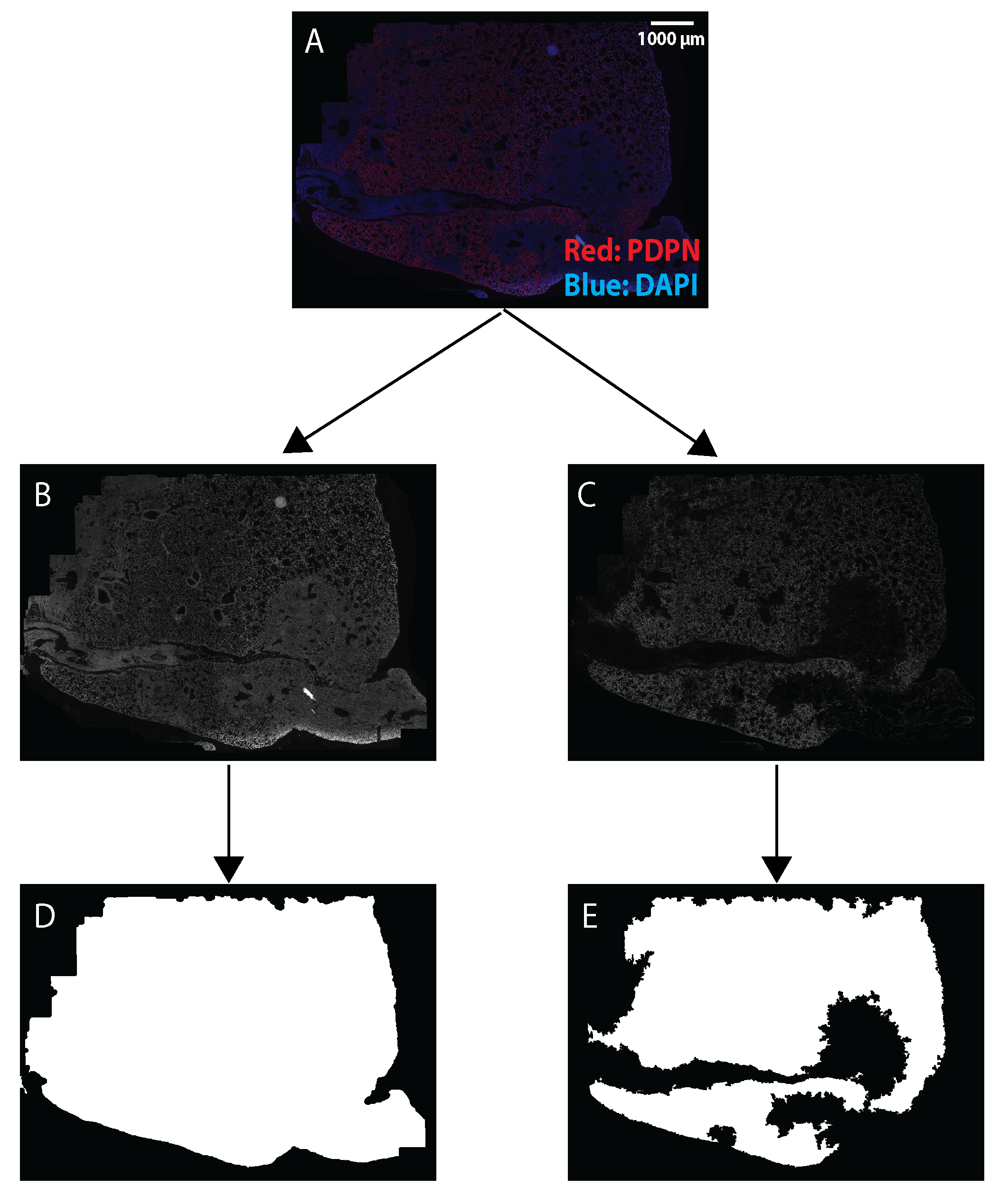

Supplement: Additional file 1: Figure S1. — Illustration of algorithm for computing AT1 coverage index A. A section of lung lobe stained with PDPN antibody (red) and DAPI (blue) B. Segmented nucleus channel from original image C. Segmented PDPN channel from original image D. Mask of total alveolar area E. Mask of PDPN + alveolar area. [file 12931_2014_116_MOESM1_ESM.tiff]
